# Exploring Alternative Sites for Glenoid Component Fixation Through Three-Dimensional Digitization of the Glenoid Vault: An Anatomic Analysis

**DOI:** 10.5435/JAAOSGlobal-D-20-00199

**Published:** 2020-12-02

**Authors:** Philip G. Ghobrial, Rahul Burra, Douglas A. Evans, Steven C. Chudik

**Affiliations:** From Loyola University Chicago Stritch School of Medicine, Maywood (Mr. Ghobrial); The University of Chicago, Chicago (Burra); the Department of Orthopaedic Surgery and Rehabilitation, Loyola University Medical Center, Maywood (Dr. Evans); Hinsdale Orthopaedics, Westmont (Dr. Chudik); Orthopaedic Surgery and Sports Medicine Teaching and Research Foundation, Westmont (Dr. Chudik); Illinois Bone and Joint Institute, Chicago (Dr. Chudik); and AMITA Health, La Grange, IL (Dr. Chudik).

## Abstract

Glenoid component loosening has remained one of the most common complications for total shoulder arthroplasty. Three-dimensional modeling of the glenoid may reveal novel information regarding glenoid vault morphology, providing a foundation for implant designs that possess the potential to extend the survivorship of the prosthesis.

**Methods:**

A three-dimensional digitizer was used to digitize the glenoids of 70 cadaveric scapulae. We identified ideal position, fit, and maximum diameter for cylinders of 5, 10, and 15 mm depths. Maximum diameter and volume were also measured at the glenoid center, and the data were compared.

**Results:**

The vault region that accommodates the greatest diameter and volume for 5, 10, and 15 mm depth cylinders were identified in the postero-inferior glenoid. Across all specimens, this region accommodated a cylinder diameter that was 24.82%, 40.45%, and 50.34% greater than that achieved at the glenoid center for 5, 10, and 15 mm depth cylinders, respectively (all, *P* < 0.0001). The location of this site remains reliable for each cylinder depth, regardless of sex.

**Discussion:**

This study presents novel findings pertaining to glenoid morphology through the analysis of a newly characterized glenoid vault region. This region has not been identified or described previously and has potential to serve as an alternative to the glenoid center for peg or baseplate fixation. Our method of vault analysis and findings may be used to guide further research regarding pathologic glenoid anatomy, providing a foundation for alternative approaches to glenoid prosthesis fixation in total shoulder arthroplasty and related procedures.

Total shoulder arthroplasty is a common surgical treatment for arthritis of the glenohumeral joint.^1^ First introduced by Emile Péan in 1893 as the platinum-rubber shoulder arthroplasty for the treatment of tuberculous arthritis, the concept of total shoulder prosthesis has undergone numerous developments over the past century.^2,3^ Dr. Charles Neer is credited with doing the first modern anatomic shoulder arthroplasty in 1974, which featured a vitallium humeral component and polyethylene glenoid component.^4^ Although more successful than previous designs, the prosthesis of Neer suffered from high failure rates because of glenoid component loosening.^3^ Despite high failure rates, this technique ultimately laid the foundation for the development of contemporary total shoulder arthroplasty devices and techniques. Today, anatomic total shoulder arthroplasty features the implantation of a stemmed metal, convex humeral component, and a concave polyethylene surface on the glenoid, replicating the anatomic relationship of the native glenohumeral joint.

Although this surgical procedure is highly successful in restoring function in the glenohumeral joint and relieving pain associated with glenohumeral degeneration, it is not free of postoperative complications.^5,6^ Prosthetic loosening accounts for approximately 39% of total shoulder arthroplasty complications, with glenoid component loosening being responsible for roughly 32% of all complications.^6^ As such, glenoid component loosening is one of the most common causes of total shoulder arthroplasty failure and is associated with symptoms such as pain, stiffness, material failure, wear, and joint instability.^5,7,8,9,10^ Further, revision surgery is often indicated in cases of glenoid component loosening.^10-12^ A clear and enduring need exists to further explore the glenoid's morphology and discover alternative methods for stabilization of the glenoid component in total shoulder arthroplasty.

Although many anatomic studies have been conducted to quantify the superficial features of the glenoid, fewer have investigated its internal spatial composition in both a qualitative and quantitative manner.^13,14,15,16,17,18,19,20,21^ Thus, digitization of the glenoid vault and its surrounding scapular structures has the potential to yield novel information about its overall morphology and potential for fixation. We hypothesize that quantification and characterization of the glenoid vault through three-dimensional (3D) digitization will ultimately provide valuable anatomic insights that could lead to improved glenoid implant design and fixation.

## Methods

Seventy preserved cadaveric scapulae were selected from the Cleveland Museum of Natural History's Hamann-Todd osteological collection. The specimens had no signs of glenohumeral degenerative disease or history of surgical procedures on the glenohumeral joint. Furthermore, the selected specimens were obtained from 37 male and 33 female cadavers ranging from 50 to 87 years old (mean, 62.8 ± 9.8). Our minimum sample size was selected in accordance with those of previously published studies investigating sexual dimorphism and glenoid size.^22,23^

**Figure 1:**
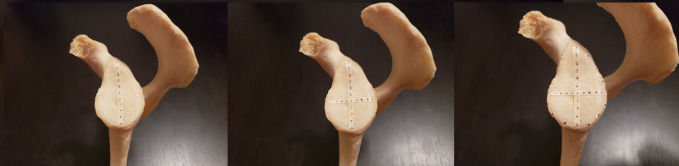
Photograph demonstrating the strip placement on the vertical axis, horizontal axis, and glenoid perimeter.

**Figure 2:**
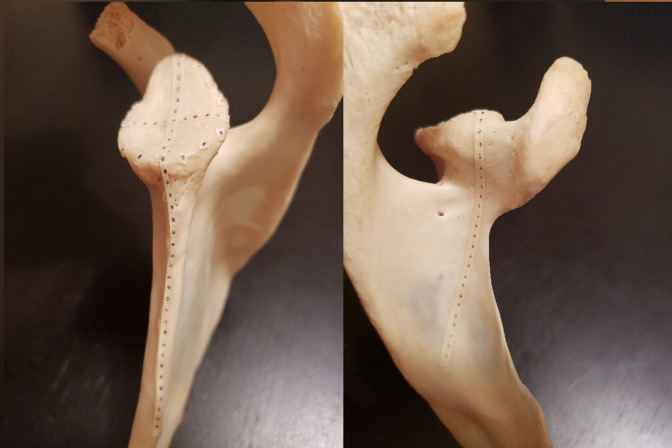
Photograph demonstrating the strip placement from the inferior and superior ends of the vertical axis.

**Figure 3:**
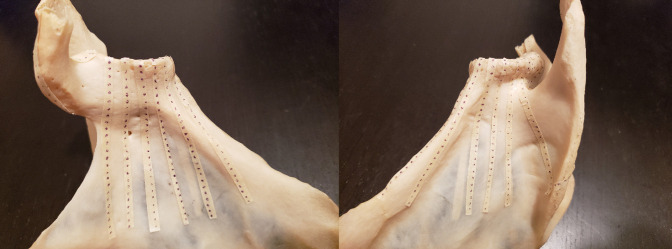
Photograph demonstrating the strip placement from each point on the perimeter of the glenoid.

**Figure 4:**
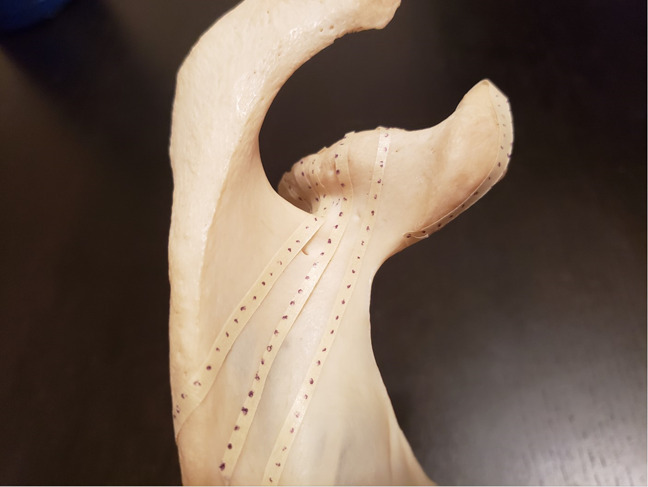
Photograph demonstrating the strip placement from each point at the perimeter of the superior-posterior glenoid.

Before data collection, a grid was created on each scapula using strips of masking tape marked with equally spaced points. First, the length of the glenoid's vertical axis was recorded in millimeters using a tape measure. A piece of tape was cut to this length and marked with 11 equally spaced points. The distance between each consecutive point was determined by dividing the length of the vertical axis by 10. The tape was then applied to the glenoid surface. The same procedure was used to label the horizontal axis. Three equally spaced points were plotted along the perimeter of the glenoid in each quadrant, forming a complete ring on the outer surface of the glenoid (Figure 1). For each quadrant, the distance between each consecutive point was approximated by measuring the length of the glenoid perimeter bordering that quadrant and dividing by 4. Next, two strips of tape were attached perpendicularly to the superior and inferior ends of the vertical axis extending onto the body of the scapula from the face of the glenoid (Figure 2), and another two strips extending onto the body were attached perpendicularly to the anterior and posterior ends of the horizontal axis. Further strips were also placed perpendicularly to the remaining points along the perimeter (Figures 3 and 4). Each strip was marked with 25 points spaced approximately 3 mm apart before application. Additional strips were added to account for areas that lacked adequate coverage.

**Figure 5:**
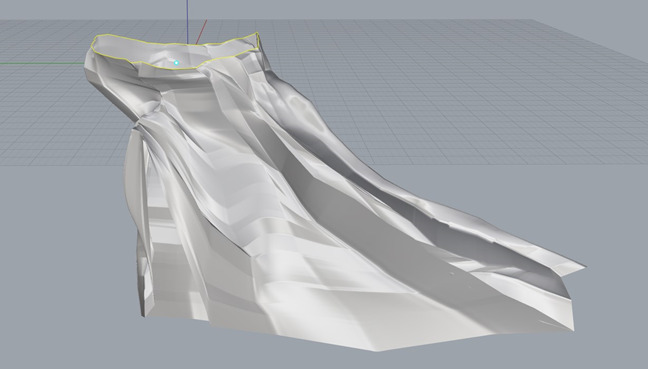
Photograph demonstrating the three-dimensional model of the glenoid and associated scapular structures.

**Figure 6:**
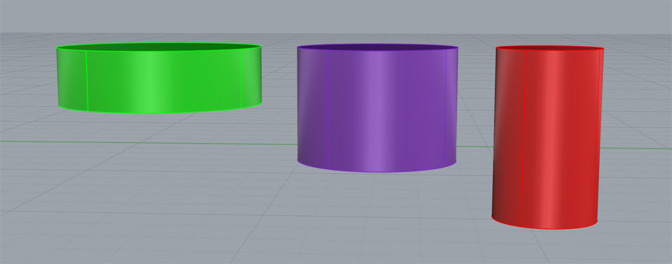
Photograph demonstrating the examples of three-dimensional cylinders used in best-fit assessment trials. Each color corresponds to a specific cylinder depth. Green = 5 mm, purple = 10 mm, red = 15 mm.

Each specimen was secured to a metal mount before data collection to prevent movement and ensure the accuracy of the digitization process. A Microscribe G2 digitizer (Immersion) was used in conjunction with Rhinoceros 3D Imaging Software (Robert McNeel & Associates). The Microscribe G2 digitizer was used to manually input the points plotted on the surface of both the glenoid and its associated scapular structures. These data were collected in Rhinoceros using the point object function; the point data collected with the digitizer were then used to generate a curve network and solid mesh reflecting the glenoid's 3D morphology (Figure 5). Identification of the vault region that accommodated the maximum diameter and volume for cylinders of 5, 10, and 15 mm depth was accomplished by manually creating 3D cylinders of varying diameter (Figure 6) and conducting multiple placement trials to identify the region of best fit (RBF). The RBF was defined as the exact position in the glenoid vault that accommodates the greatest diameter for each cylinder depth without breakage through the surrounding cortical surface bone. When the RBF was identified for a given cylinder depth, placement trials were repeated by the investigator twice to ensure the reproducibility and accuracy of the result. The intraclass correlation coefficient was measured using the data collected from these measurements. The cylinder placement trials were conducted in a manner whereby each cylinder was both level with and perpendicular to the articular surface at the glenoid center.

**Figure 7:**
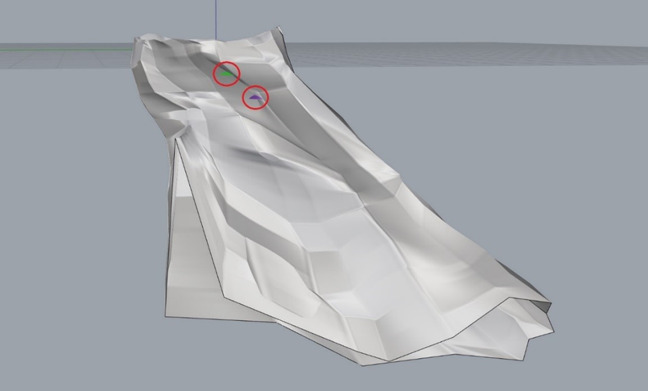
Photograph demonstrating an example of cortical breakage, or a “bad fit.” This image depicts cortical bone destruction induced by two different cylinder types. The green and purple protrusions within the red circles signify the inferior aspect of each cylinder breaking through the surrounding cortical bone.

**Figure 8:**
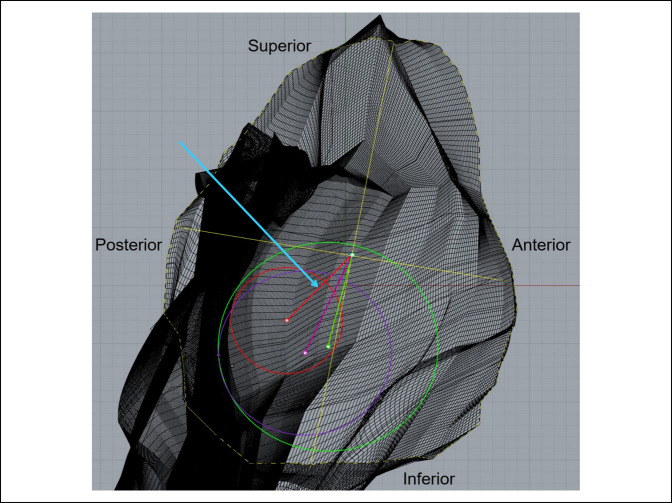
Photograph demonstrating the top-down view of the glenoid vault. The colored circles represent the varying cylinder depths: green = 5 mm, purple = 10 mm, red = 15 mm. The colored dots contained within each circle represent the center of each respective cylinder. The cyan point depicts the glenoid center. The colored lines from the glenoid center to the cylinder centers (red, purple and green) represent the distance between the respective points. The blue arrow is pointing to the distance measurement between the glenoid center and the center of the 15 mm depth cylinder.

These cylinders were fit within the body of the glenoid at varying locations in accordance with the glenoid's capacity to accommodate their differing depths. A bad fit was defined as a cylinder diameter and/or placement that resulted in breakage through the cortical surface bone (Figure 7). When a bad fit occurred, the trial would be repeated in a different vault region. If a bad fit occurred throughout the entirety of the vault, cylinder diameter was decreased by 0.1 mm, and the procedure was repeated. When the position that accommodated the greatest cylinder diameter for a given depth without breakage through the cortical surface was identified, the location of the point where the central axis of the cylinder intersects the glenoid articular surface (center of cylinder) relative to the center of the glenoid's articular surface (glenoid center) was measured. This was accomplished by measuring the distance from the glenoid center to the central axis of the cylinder on the glenoid articular surface (Figure 8). The glenoid center was designated as the intersection of the midpoints of the vertical and horizontal axes on the glenoid articulating bony surface.

**Figure 9:**
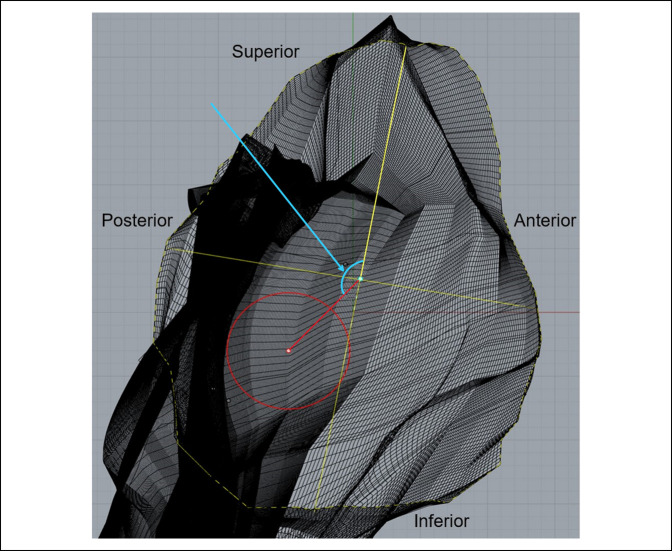
Photograph demonstrating the top-down view of the glenoid vault depicting angle measurement. This image depicts the method by which angle measurements were taken. The angle between the superior aspect of the vertical axis and the cylinder center was measured for each cylinder type. The blue arrow is pointing to the angle measurement.

**Figure 10:**
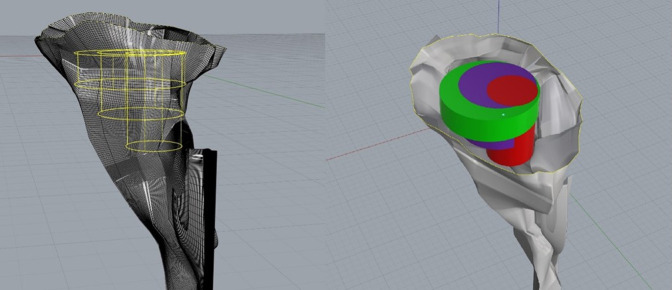
Photograph demonstrating the view from superior end of glenoid showcasing best-fit cylinder placement.

The position of the center of the cylinder was described by the distance from the glenoid center and the angle made between the 12 O'clock position of the vertical axis and a line from the glenoid center to the center of the cylinder (Figure 9). Figure 10 represents an example of final cylinder placement in the RBF after completion of the aforementioned testing phase. Cylinder volumes for each successful trial were also measured using Rhinoceros 3D geometric analysis functions. Placement trials for cylinders of 5, 10, and 15 mm depth were then conducted at the glenoid center to identify the maximum diameter and volume for each cylinder depth.

The data yielded from the RBF and glenoid center were statistically analyzed using SPSS Statistics for Windows (IBM). The data were statistically compared using the paired sample *t* test and repeated measures analysis of variance. For comparisons between sexes, the independent samples *t* test was used. Alpha was set at 0.05. Furthermore, model construction, cylinder fitting, and data collection were done by a single investigator. Intrarater reliability was assessed through calculation of the intraclass correlation coefficient in SPSS based on a single rater, absolute agreement, two-way mixed effects model.

## Results

Data collected for the 37 male specimens revealed that for 5, 10, and 15 mm depth cylinders situated in the RBF, the average maximum diameter was 22.35 ± 2.93, 17.21 ± 3.48, and 13.32 ± 3.14 mm, respectively. The center of the 5 mm depth cylinder in the RBF was located at an average angle of 171.24 ± 18.11° in the postero-inferior quadrant, at a distance of 6.00 ± 1.92 mm from the glenoid center. This 5 mm depth cylinder possessed, on average, a volume of 1995.37 ± 534.29 mm^3^. The center of the 10 mm depth cylinder in the RBF was located at an average angle of 174.06 ± 15.12° in the postero-inferior quadrant at a distance of 7.76 ± 2.84 mm from the glenoid center. The average 10 mm depth cylinder volume was 2418.60 ± 999.18 mm^3^. The center of the 15 mm depth cylinder in the RBF was located at an angle of 169.38 ± 21.67° in the postero-inferior quadrant and 7.81 ± 3.64 mm from the glenoid center. The average 15 mm depth cylinder volume was 2237.56 ± 1064.42 mm^3^. The ideal location for achieving maximal cylinder diameter and volume, regardless of cylinder depth, was found in the postero-inferior region of the glenoid for male specimens. No significant difference was found between the angles for the position of each cylinder depth (all pairs, *P* > 0.05). However, the difference in distance from the glenoid center was statistically significant for the 5 and 10 mm depth cylinders (*P* < 0.0001), as well as for the 5 and 15 mm depth cylinders (*P* < 0.0001). No significant difference was noted in the distance from the glenoid center between the 10 and 15 mm cylinders (*P* = 0.647).

The 33 female specimens, on the other hand, yielded average maximum diameters of 16.55 ± 1.96, 11.15 ± 1.83, and 8.48 ± 1.91 mm for 5, 10, and 15 mm depth cylinders, respectively. The center of the 5 mm depth cylinder in the RBF was located at an angle of 171.31 ± 11.67° in the postero-inferior quadrant at a distance of 5.39 ± 1.78 mm from the glenoid center. The average volume for the 5 mm depth cylinder was 1050.69 ± 226.53 mm^3^. The center of the 10 mm depth cylinder in the RBF was located at an average angle of 174.32 ± 11.30° in the postero-inferior quadrant and 7.45 ± 3.04 mm from the glenoid center. The average 10 mm depth cylinder volume was 1001.53 ± 320.14 mm^3^. The center of the 15 mm depth cylinder in the RBF was located at an angle of 174.43 ± 13.08° in the postero-inferior quadrant and a distance of 8.37 ± 3.49 mm from the glenoid center. The average 15 mm depth cylinder volume was 863.66 ± 345.47 mm^3^. No significant difference was noted between the angles for each cylinder depth (all pairs, *P* > 0.05). As seen in the male specimens, the difference in distance from the glenoid center was statistically significant for the 5 and 10 mm depth cylinders (*P* < 0.0001), as well as for the 5 and 15 mm depth cylinders (*P* < 0.0001). No significant difference was found in the distance from the glenoid center between the 10 and 15 mm cylinders (*P* = 0.148).

On average, the ideal location for achieving maximal cylinder diameter, regardless of cylinder depth, was found in the postero-inferior region of the glenoid for both male and female specimens. The posterior-inferior region was consistent with an angle measurement between 90 and 180°. The 95% confidence intervals for angle measurements for 5, 10, and 15 mm depth cylinders were (163.27–174.85), (169.58–178.37), and (160.77–170.15), respectively. The RBF was located in the postero-inferior region for most, but not all subjects. 74.3%, 61.4%, and 72.0% of the 5, 10, and 15 mm depth cylinders, respectively, were located in the posterior-inferior region. The remainder of the cylinders for each cylinder depth were located in the anterior-inferior region. The average angle measurements for the 5, 10, and 15 mm cylinders in the anterior-inferior region were 184.34 ± 2.85, 187.55 ± 6.38, and 191.33 ± 14.04, respectively. All specimens with a RBF in the anterior-inferior region also accommodated cylinders in the posterior-inferior region that were significantly greater in diameter and volume compared with cylinders at the glenoid center. However, because these cylinders did not meet our predefined criteria for the RBF, the associated data were not included in our analysis. Furthermore, the angle and distance from the glenoid center did not differ significantly between male and female specimens for 5 (angle, *P* = 0.450; distance, *P* = 0.325), 10 (angle, *P* = 0.885; distance, *P* = 0.827), or 15 (angle, *P* = 0.511; distance, *P* = 0.515) mm cylinders. The average spatial coordinates of the RBF for each cylinder depth remained consistent, regardless of sex.

These data were then compared with the maximum diameter and volume measurements taken at the glenoid center. For the male specimens, the glenoid center accommodated maximum cylinder diameters of 17.94 ± 3.33, 12.64 ± 3.89, and 9.37 ± 3.95 mm for 5, 10, and 15 mm depth cylinders, respectively. Comparatively, the RBF accommodated a maximum cylinder diameter that was, on average, 24.65%, 36.16%, and 42.16% greater for the 5, 10, and 15 mm depth cylinders, respectively. The glenoid center accommodated a maximum cylinder volume of 1306.05 ± 473.51, 1370.19 ± 832.82, and 1213.39 ± 917.41 mm^3^ for the 5, 10, and 15 mm depth cylinders. Comparatively, the RBF accommodated a maximum cylinder volume that was, on average, 52.78%, 76.52%, and 84.41% greater for the 5, 10, and 15 mm depth cylinders, respectively. The difference in maximum cylinder diameter and volume between the RBF and glenoid center was statistically significant for all cylinder depths (all, *P* < 0.0001).

For the female specimens, the glenoid center accommodated maximum cylinder diameters of 13.04 ± 2.64, 7.60 ± 3.01, and 5.24 ± 2.73 mm for 5, 10, and 15 mm depth cylinders, respectively. Comparatively, the RBF accommodated a maximum cylinder diameter that was, on average, 26.92%, 46.71%, and 61.83% greater for the 5, 10, and 15 mm depth cylinders, respectively. The glenoid center accommodated a maximum cylinder volume of 694.24 ± 268.65, 523.08 ± 415.72, and 408.82 ± 425.67 for the 5, 10, and 15 mm depth cylinders, respectively. Comparatively, the RBF accommodated a maximum cylinder volume that was, on average, 51.34%, 91.47%, and 111.26% greater for the 5, 10, and 15 mm depth cylinders, respectively. The difference in maximum cylinder diameter and volume between the RBF and glenoid center was statistically significant for all cylinder depths (all, *P* < 0.0001). Compared with female specimens, male specimens had significantly greater maximum cylinder diameter and volume for each cylinder depth at the RBF (all, *P* < 0.0001). Summaries of key findings for male and female specimens are contained in Supplemental Tables 1 (http://links.lww.com/JG9/A104) and 2 (http://links.lww.com/JG9/A105), respectively.

The single measures intraclass correlation was 1.00 for diameter, angle, and distance measurements for all cylinder depths. The 95% confidence interval was (1.00–1.00) for cylinder diameter, angle, and distance measurements for all depths, indicating perfect reliability. No variation was noted between the statistical output of parametric and nonparametric tests for nonparametric data sets.

## Discussion

Characterization and quantification of the glenoid vault through 3D digitization has the potential to yield novel information about the glenoid's vault morphology and potential room for a prosthetic implant. In addition to its ability to accurately capture the dimensions that comprise the anatomical architecture of both the glenoid vault and its neighboring scapular structures, 3D digitization provides a platform that can analyze the glenoid's spatial composition with precision. The 3D digitization, model creation, and data analysis done in this study yielded results that are valuable to our understanding of glenoid morphology and also provide insights for further innovation in surgical technique and glenoid implant design, validating our hypothesis.

Previous studies have used cadaveric specimens, radiographs, 2D and 3D CT, magnetic resonance imaging scans, and computer models to characterize and quantify the glenoid's morphology.^24,25,26,27,28,29,30,31,32,33,34,35^ These studies have described the glenoid's superficial features and internal characteristics such as the depth, width, and bone density within varying regions of the glenoid vault. For example, analysis of Sharma et al^36^ of the glenoid through CT imaging and 3D computer modeling yielded information pertaining to glenoid version, regional vault depth, and regional bone density. In their study, a point named the “circle center” was characterized in the inferior glenoid, which marked the location that possessed the greatest vault depth. The location of the circle center is similar to that of the RBF defined in our study. However, Sharma et al did not identify specific coordinates for this point. Instead, they approximated the inferior glenoid boundary using a circle, whose center they termed the “circle center.” Our study provides specific coordinates for the RBF located within a similar region while also supplying data regarding the maximum cylinder diameter and volume that can be achieved at varying depths.

Furthermore, combining bone density and glenoid vault data, Sharma et al concluded that the circle center or mid-glenoid should be prioritized for glenoid component fixation. However, they did not identify the maximum diameters that could be accommodated at varying depths, which would be useful for achieving maximal area of fixation in the available bone of the glenoid vault. Our study quantified the maximum cylinder diameter and volume that can be achieved at a depth of 5, 10, and 15 mm within the RBF and the glenoid center, although also showing that the maximum diameters and volumes are significantly greater at the RBF compared with the glenoid center for all cylinder depths. If interpreted in tandem with the findings of Sharma et al, our data suggest that the RBF may be the preferred fixation site when considering maximum cylinder diameter, vault depth, and bone density.

On average, the center for each cylinder type in the RBF was located in the posterior-inferior quadrant of the glenoid. For most specimens, other regions of the glenoid either supported a significantly smaller cylinder diameter or produced cortical bone penetration because of their inability to accommodate the full depth of the cylinders. The coordinates of the RBF remained consistent for each cylinder depth, regardless of sex (all, *P* > 0.05) despite the significant difference in cylinder diameter and volume between male and female specimens (*P* < 0.0001). The RBF also remained consistent despite variations in specimen size. These findings may be indicative of patterns in glenoid vault morphology that persist, regardless of specimen sex or size. This observation aligns with the findings of Codsi et al,^24^ who determined that the internal shape of the glenoid vault in a sample of skeletally mature scapulae featured uniform morphology despite a wide variation in the gross scapular size. Furthermore, a significant difference in the center-RBF distance between 5 to 10 mm and 5 to 15 mm cylinder pairs was also found for both sexes (*P* < 0.0001), with no significant difference in the glenoid-RBF distance between the 10 and 15 mm cylinders (male, *P* = 0.647; female, *P* = 0.148). This suggests that for 5 mm depth cylinders, the distance to the RBF from the glenoid center is significantly smaller than that required for 10 or 15 mm depth cylinders. This information may help guide future studies that seek to further investigate this region using preset cylinder depths.

Perhaps most importantly, our study revealed that this newly characterized region possesses a greater spatial capacity than the default site for glenoid component fixation, the glenoid center. Across all specimens, this new region accommodated a maximum cylinder diameter that was, on average, 24.82%, 40.45%, and 50.34% greater than that achieved at the glenoid center for 5, 10, and 15 mm depth cylinders, respectively. The differences in diameters achieved was statistically significant for all cylinder depths, regardless of sex (all, *P* < 0.0001). Furthermore, cylinder volume for 5, 10, and 15 mm depth cylinders was also, on average, 50.71%, 81.87%, and 94.65% greater at the RBF across all specimens. Again, the difference in volume achieved was statistically significant for all cylinder depths, regardless of sex (all, *P* < 0.0001).

To our knowledge, the RBF described in this study has not been identified or described previously. Furthermore, this is the first time that a region other than the glenoid center has been characterized and quantified in a manner that showcases potential spatial superiority over the glenoid center for the purpose of glenoid component fixation. The method of glenoid vault analysis used this study has also not been done in this manner previously and may assist in determining the viability of varying angles and locations for post, peg, or screw fixation placement based on existing data pertaining to the cortical bone morphology, thickness, and density.

Although our study provides a foundation for further investigation and potential innovation, additional research into this newly characterized region must be conducted in pathologic glenoids before conclusions regarding its potential because a fixation site can be drawn. The importance of conducting further research in pathologic glenoids is evidenced by the findings of previous studies investigating morphological alterations in this specimen population. For example, Frankle et al^37^ reported that 62.5% of glenoids were morphologically normal and 37.5% were abnormal in 216 patients who had undergone reverse shoulder arthroplasty. After the abnormal glenoids were further stratified based on erosion sites, it was found that 17.6% of all glenoids possessed posterior erosions that had a significant effect on both anatomical structure and surgical factors. Given that our RBF was located in the posterior region of the glenoid, it is possible that our findings may not be fully reproducible in certain samples of pathologic glenoids with posterior bone loss and associated morphologic changes.

Some additional limitations exist to our study. First, our method of glenoid vault analysis has not been used previously. Given the absence of literature investigating the glenoid vault in the manner outlined in our study, currently no data are available for comparison. More research must be conducted with varying methods of 3D modeling and analysis to validate the findings of our study. In addition, the glenoids used in this study were skeletally normal, lacking the acquired degenerative or developmental deformities often seen clinically. Because of the altered structural characteristics associated with pathologic glenoids, we cannot completely extrapolate our data to all clinical situations. Future studies should consider examining these differences in morphology among different glenoids to determine whether the RBF remains consistent in the presence of typical patients with glenohumeral arthritis. However, the data obtained through our study can be used as a foundation to guide future studies of similar scope.

Finally, our study did not evaluate regional bone density. Bone density plays an important role in the fixation of the glenoid component and may display significant regional variation in pathologic glenoids. Simon et al^38^ reported that glenoid subchondral bone density (SBD) patterns varied in men who underwent total shoulder arthroplasty. Their study found that SBD varied according to whether the glenoid possessed a concentric or eccentric wear pattern. In the eccentric group, the SBD distribution was inhomogeneous and mineralization was greatest in the posterior zone. However, in glenoids with concentric wear patterns, the SBD distribution was homogeneous, with greater mineralization in the central zone compared with the posterior, anterior, and superior zones.^38^ Thus, future research into the RBF of pathologic glenoids should also take bone density into account if conclusions regarding fixation potential are to be made.

Despite these limitations, this study presents novel findings pertaining to glenoid morphology through the analysis of a newly characterized glenoid vault region. This region has not been identified or described previously and has potential to serve as an alternative to the glenoid center for peg or baseplate fixation. Our method of vault analysis and findings may be used to guide further research regarding pathologic glenoid anatomy, providing a foundation for alternative approaches to glenoid prosthesis fixation in anatomic total shoulder arthroplasty and related procedures.
